# Supplementary material for: Are carbon emissions trading and green financial instruments synergistic? -Comprehensive quantitative research based on content analysis
Source: PLoS One. 2024 Mar 7;19(3):e0298601. doi: 10.1371/journal.pone.0298601 (PMC10919601; doi:10.1371/journal.pone.0298601)
Supplement: S1 Data — (ZIP) [file pone.0298601.s001.zip › Data/地级市Z_F工作报告.zip!/║ú─╧/║ú┐┌╩╨/└┤╫╘╣1⁄2╓┌║┼í╛┬φ┐╦╩2╛▌═°í┐_2007.docx]

各位代表：

现在，我代表市人民政府，向大会作政府工作报告，请予审议，并请各位政协委员和其他列席人员提出意见。

一、过去四年的工作回顾

2003年以来，在省委、省政府和市委的正确领导下，我市高举邓小平理论和“三个代表”重要思想伟大旗帜，坚持以科学发展观统领经济社会发展全局，审时度势，开拓创新，基本完成了“十五”计划任务，实现了“十一五”规划的良好开局。

（一）国民经济持续快速发展，产业结构进一步优化

经济持续快速发展。从2002年到2006年，全市生产总值从204.59亿元增加到350.06亿元，年均增长13.0%，连续九年保持两位数增长,人均生产总值从12816元增加到19980元；固定资产投资从93.57亿元增加到158.23亿元，四年累计完成投资522.28亿元，年均增长14%；财政总收入从32.5亿元增加到70.08亿元，翻了一番以上，年均增长21.2%；城市居民人均可支配收入从8004元增加到10712元，年均增长7.6%；农民人均纯收入从3086元增加到4139元，年均增长7.6%。产业结构不断优化，三次产业结构从2002年的8.4:27.3:64.3调整到7.4:29.2:63.4。2005年获全省经济社会综合考核一等奖。

农业平稳发展。农业产业结构得到优化，农业科技“110”进一步推广，农业信息化水平不断提高，畜牧业、种植业、渔业、花卉产业发展较快，农业总产值从2002年的27.39亿元增加到43.07亿元。其中，畜牧业产值占农业总产值的比重从31.9%提高到36.9%，生猪产量逐年提高，从依靠岛外供应发展到自给再出岛并进入了香港市场；水产品2006年出口总量达3.2万吨，创汇1.09亿美元，分别比2003年增长77.8%和196.7%；四年完成造林1.1万公顷，城乡绿网建设得到加强。我市2005年被评为“全国农业产业化工作先进单位”和“中国果菜龙头市”，农田水利建设连续三年获得省评比一等奖，森林防火工作连续四年受到省通报表彰。

工业加快发展。工业向保税区、高新区和海马汽车工业园等园区集中发展。2006年园区工业总产值占全市规模以上工业总产值的58.7%。产业特色日趋显现，形成以汽车制造、生物制药、化纤纺织、食品饮料、热带农产品深加工为主的产业结构，五大支柱产业产值占全市规模以上工业总产值的80.1%。企业规模不断壮大，三星光缆、盛之业、齐鲁制药、万特制药等一批大企业建成投产，目前全市规模以上企业达到219家，总产值年均增长21.1%。经济效益明显提高，2006年全市工业总产值达到302.45亿元，工业增加值达到79.81亿元，利润总额达到17.45亿元，分别比2002年增长1.15倍、1.06倍和1倍。

旅游业健康发展。紧紧围绕打造“阳光海口·娱乐之都”、建设全国热带滨海度假休闲胜地的目标，编制《海口市旅游业发展总体规划纲要》，出台扶持航空客运、旅行社和景区（点）发展的相关政策，加大旅游宣传促销，开展对旅游市场“四黑”综合整治，推进雷琼世界地质公园海口园区、海南野生动植物园等重点项目建设，策划打造一批品牌旅游景区(点)，成功举办海口旅游商品交易会、海南岛欢乐节、冼夫人文化节、高尔夫球赛等一系列活动，促进旅游业健康发展。全市接待国内外过夜旅游者从2002年的382.09万人次增加到536.52万人次，旅游总收入从29.99亿元增加到51.23亿元。

商贸业进一步发展。全面完成商业网点规划工作，商业网点规模不断扩大，南亚广场、宜欣广场等144个大中型商业网点开业，新增营业面积101.35万平方米，总投资18.8亿元。肯德基、麦当劳等餐饮业连锁经营规模持续扩大，提升了我市商贸业的业态；家乐福、大润发等多个国内外知名企业的进驻，进一步优化了我市的商业结构，促进了我市商业规范管理。社会消费品零售总额从2002年的92.64亿元增加到161.35亿元。2006年全市外贸口岸进出口总额13.43亿美元，其中，出口5.4亿美元，比2002年翻了一番；地产品出口3.5亿美元，占全市外贸口岸出口总额的70%。

房地产业稳健发展。积极落实国家宏观调控政策，努力处置积压房地产，四年共有234宗、694万平方米停缓建工程复工，基本完成处置任务。四年共完成房地产投资169.09亿元，建成宝安·江南城、长信海岸水城等一批精品小区，形成建设、销售、需求的良性循环态势，呈现岛内岛外购房各占一半的良好局面。商品房销售面积从2002年的56.76万平方米增加到141.64万平方米，增长1.5倍。

（二）社会事业加快发展，人民群众得到更多实惠

社会事业加快发展。具有世界领先水平的维瑅瑷(VTI)生物研究院落户我市，实现了我市生物技术研发领域的历史性突破。2006年全市高新技术产业产值达178亿元，比2002年增长4.8倍。四年全市教育总投入达42.5亿元，新建改建各类学校319所，中小学办学条件得到很大改善；实施277所农村中小学现代远程教育工程，率先在全省落实了“两免一补”政策，城乡教育差距不断缩小；职业、成人教育得到加强，民办教育健康发展，基础教育继续在全省保持领先水平，“两基”覆盖率达100%。文体事业蓬勃发展，歌舞诗《达达瑟》等一批作品分获国家、省级奖，成功举办全国健美操锦标赛、第二届世界太极拳健康大会、《城市之间》中国区总决赛等一系列大型活动，并首次获《城市之间》国际版入场券；积极开展广场文体活动和文体下乡活动，大致坡镇的琼剧文化发展得到中宣部的肯定。新建扩建市妇幼保健院、市医院、市中医院、市120急救中心和镇卫生院，公共卫生体系和公共卫生应急网络不断完善，全力做好“非典”等突发性疾病防控工作，应急救治能力进一步增强；全市社区卫生服务覆盖率达到80%；新型农村合作医疗覆盖面不断扩大，全市已有47.79万农民自愿参加，参合率达到95%。群众性精神文明创建活动广泛开展，被评为全国创建文明城市工作先进城市；法制宣传教育深入扎实，被评为全国“四五”普法先进城市；计生、双拥、民族进步、妇女儿童工作成效显著，连续三年被评为全省人口与计划生育目标管理先进单位，实现全国双拥模范城“五连冠”的目标，获全国民族团结进步模范集体和全国妇女儿童权益贡献奖。统计、审计、人防、气象、档案、地方志等各项工作都取得新的成绩。

为民办实事成效显著。四年来向社会公开承诺的61件为民办实事事项基本兑现，群众关心的上学难、看病难、住房难、行路难、打官司难等民生问题得到较大缓解。四年共投入1.31亿元用于中小学危房改造，获全省中小学危房改造攻坚工程一等奖。对全市义务教育阶段学生99.25万人次免杂费9566.2万元，对义务教育阶段农村学校学生和城区特困家庭子女26万人次免教科书费1675.9万元，为2054名农村贫困家庭寄宿生补助生活费81.77万元。累计新增就业9.9万人，其中下岗失业人员再就业2.8万人，城镇登记失业率控制在4%以内。城乡低保实现全员覆盖，做到应保尽保，差额补助标准不断提高，2006年全市有51万人次进入低保范围。设立农民工工伤医疗救助资金，制定城镇特困人员医疗救助办法，对农民工和城乡特困家庭提供医疗救助；维护社会弱势群体的合法权益，为经济困难的本市居民和外来务工人员提供法律援助。修建662套廉租房，解决了一批城市特困居民的住房问题。完成了669条小街小巷改造及1106条道路路灯安装。

社会政治稳定。加强社会治安综合治理，大力打击“两抢一盗”和毒品犯罪，刑事案件高发势头得到有效遏制，发案率逐年下降,群众的安全感普遍增强。坚决打击“法轮功”等邪教组织，严密防范境内外敌对势力的渗透破坏活动。建立健全社会矛盾调处机制，妥善处理各种矛盾纠纷和群体性上访事件。深入开展食品、药品市场等专项整顿。加强安全生产监管，建立公共突发事件应急处理机制，颁布实施《海口市人民政府突发公共事件总体应急预案》及26个专项预案，应急事件的处置能力得到增强，社会政治保持稳定。

（三）城市功能不断完善，宜居城市建设成效明显

城市基础设施建设力度加大。完成了城市总体规划的修编，制定了长流起步区、东海岸等一系列片区规划及专项规划，抓好小城镇总体规划的编制。城市建设由扩大规模向拓展空间与完善功能并举发展。四年共投入37.31亿元，组织实施55项道路、桥梁工程，琼山大道、世纪大桥等一批市政项目建成通车，绕城公路、长秀片区路网工程等一批项目开工建设。城市框架全面拉开，市区“八纵四横”的路网基本形成，城市基础设施不断完善。城市化率由2002年的53.3%提高到2006年的59.5%。

人居环境建设加快推进。加大环境保护，实施颜春岭垃圾填埋场垃圾渗透液处理工程，建设白水塘垃圾中转站和一批小型垃圾转运站，实施龙塘水源点保护、“一园两湖”改造和市中心区污水截流并网工程等一系列环保工程。2006年安排3000万元，基本完成凤翔路、琼州大道的街景立面改造。启动城市管理综合执法，开展城市环境综合整治，市容“六乱”现象得到有效遏制，四年共拆除违法建筑96.6万平方米，300多亩乱坟岗变成了绿地。四年共新增改造绿地面积820公顷，城市建成区绿化覆盖率达39.5%，全市森林覆盖率达35%。城市空气质量继续保持国家Ⅰ级水平，获中国人居环境奖。

（四）镇域经济加快发展，社会主义新农村建设开局良好

镇域经济发展加快。坚持“多予、少取、放活”的方针，全面取消农业特产税、农业税，实行良种和农机具补贴等一系列政策，从政策上扶持镇域经济发展。启动永兴、新坡、红旗、大致坡四个重点镇的规划建设，引导家具、花卉、建材、服装等部分专业市场向近郊城镇转移，支持各镇发展非农产业，形成了石山、红旗、大致坡等一批特色产业镇。打工经济成效显著，农村劳动力转移就业2.9万人次，农民收入稳步增长。

农民生产生活条件明显改善。农业基本建设得到加强，共投入8050万元，完成一批田洋整治和水库的除险加固，改善了农业的生产条件。加强乡村道路建设，共完成161条、655公里的乡村公路建设工程，实现了村村通公路的历史性突破。全面解决全市农村人畜饮水难问题，同时启动农村安全卫生饮水建设，全市农村自来水覆盖率达到了92%。投入2400万元完成32幢防洪楼建设，可容纳灾民3.1万人。

文明生态村建设成效显著。加大投入，全面推进文明生态村建设，四年市财政共安排1460万元、各区财政投入2090万元和驻军单位筹集500多万元以及群众自筹资金812万元，用于文明生态村建设。通过发展沼气解决畜禽场污染、村庄环境卫生、农民节支增收等六大问题，成为文明生态村建设的一大亮点。到2006年底，全市已建成文明生态村812个，占全市自然村总数的38%。2006年，我市被评为省文明生态村建设工作示范市，我市也因此获得第二届中国全面小康论坛“中国十大政府创新典型”奖。认真贯彻落实我市《关于推进社会主义新农村建设的实施意见》，有力地推进社会主义新农村建设。

（五）改革开放进入新阶段，经济发展的活力显著增强

改革取得新进展。完成了行政区划调整，拓展了我市发展的空间。加快理顺市、区两级行政管理体制，把规划、建设、水务、环卫等部分行政事项下放各区。稳步推进财政体制改革，基本完成政府收支分类改革，积极推行部门预算、国库集中支付改革。成立市政府投资项目管理中心和工程项目审计中心，对社会口政府投资项目全面推行“代建制”，规范政府投资行为。完成国资委、安全生产监督管理局等机构的设置和调整，成立市城市管理行政执法局，实施城市管理综合执法；调整了海口保税区、高新区的管理体制，成立了西海岸开发建设管委会。完成椰树集团的改制。整合公共资源，组建旅游控股、水务集团、城投公司，完成海南港航控股公司的接收工作。积极引导和扶持民营经济发展，民营经济比重达50%以上。搭建融资平台，缓解中小企业融资难问题。

对外开放取得新成就。积极实施“双大互动”和“大企业进入，大项目带动”发展战略，广泛参与泛珠三角等区域合作，开展形式多样的推介会、招商会等活动。三星光缆公司等6家世界500强企业和新世界等国内外有实力的企业相继进驻海口。四年共引进209个项目，投资总额77.06亿元人民币，新设立外资企业370家，实际外商直接投资14.23亿美元，其中，2006年达到4.47亿美元，比2002年增长82.8%。国际交流与合作日趋活跃，国际友好城市从原有的5个增加到8个，侨务工作也取得新进展。

（六）政府自身建设不断加强，执行能力明显提高

行政行为不断规范。自觉接受人大的法律监督、工作监督和人民政协、民主党派的民主监督。四年来，市政府及各部门共办理人大议案和建议997件，政协提案1260件。全力营造勤政为民的政务环境，正式启用海口市政务服务中心，进入中心的行政审批事项占全部审批事项的74.5%，行政许可事项按期办结率达到99%；建立市长接待日制度和行政首长问责制，完善政府管理流程，规范行政行为，提高办事效率，政府常务会议议定事项办结率由30%左右提高到90%以上。大力推进民主科学决策，严格贯彻《市直单位领导班子重大问题议事规则》，建立决策责任制度，增强一把手集体决策意识，规范决策程序，提高决策水平。建立社会公示与社会听证制度和政府新闻发言人制度，接受舆论和群众监督。组织监督专员和特邀监察员走访百家企业听取意见，开展万人评议政府机关问卷调查，有力地促进了政府机关效能建设，提升了政府的公信度。完善政府规章，设立市政府法律顾问团和市政府首席法律顾问，依法行政意识不断增强，行政执法水平明显提高。认真开展保持共产党员先进性教育等学习教育活动，政府工作人员的宗旨意识、公仆意识和服务意识进一步增强。

廉政建设取得新成效。认真贯彻落实中央、省委和市委关于党风廉政建设的各项部署，出台《海口市政府廉政建设的若干规定》及《实施意见》和《海口市行政首长问责制暂行规定》，加强机关的党风廉政建设，不折不扣地执行中央关于廉洁自律的各项规定，从严查处各种违纪违法行为。加强工程招标、产权交易、政府采购等关键环节的监管，搞好领导干部经济责任审计。全面开展自查自纠、查办案件和建立健全长效机制等工作，治理商业贿赂，形成以党风廉政建设促发展的良好局面。

各位代表！过去四年是海口历史上发展又好又快的四年，是产业发展力度较大、民生问题解决较好、城市面貌变化较快、人民生活明显改善的四年。这些成就，归功于省委、省政府和市委的科学决策与正确领导，归功于市人大及其常委会、市政协的有力监督和大力支持，归功于全市人民的辛勤奉献和团结奋斗。在此，我谨代表海口市人民政府，向所有关心、支持和参与海口发展的广大工人、农民、知识分子、企业界人士、驻市部队、武警官兵、政法干警、中央和省驻海口单位、各民主党派、工商联、无党派人士、各人民团体、离退休老同志和海内外朋友，表示衷心的感谢和崇高的敬意！

在充分肯定成绩的同时，我们也清醒地认识到经济社会发展中存在的一些困难和问题：一是经济发展的结构性矛盾依然突出，农业基础不强，工业化水平低，特色产业链条短，附加值不高，品牌效益和规模优势还不够明显。二是社会投资不够活跃，融资渠道单一，城镇基础设施建设、经济社会发展受到资金短缺的严重制约。三是就业压力较大，城乡居民收入水平较低，农民增收难度大，部分群众生活还比较困难。四是社会事业发展仍然滞后，公共服务水平低，文化体育基础设施薄弱，群众行路、饮水、上学、就医等方面的困难和问题还较多。五是因职工下岗、城镇拆迁、农民失地、涉法涉诉等引发的不稳定问题仍然突出。这些困难和问题需要政府在今后的工作中认真加以解决。

二、新一届政府工作目标和主要任务

根据党的十六届六中全会精神和市第十一次党代会精神,以及我市“十一五”规划总体要求，新一届政府工作的指导思想是：坚持以邓小平理论和“三个代表”重要思想为指导，全面贯彻落实科学发展观，突出大特区省会体制的“特”字，进一步优化经济结构，着力转变增长方式，推进改革开放和自主创新，加强资源节约和环境保护，注重解决民生问题和促进社会事业发展，推动经济社会又好又快发展，努力把我市建设成为繁荣富裕、环境优良、社会和谐的省会城市。

我们将着力打造环境特色、产业特色、体制特色、城市特色和文化特色，努力实现“十一五”规划提出的“三个翻番、两个突破”和市第十一次党代会提出的经济社会发展目标，为构建和谐海口奠定坚实的基础。今后五年预期的主要经济社会发展指标是：到2011年，全市生产总值达到565亿元，年均增长11%；财政总收入达到114亿元，年均增长11%；城镇居民人均可支配收入15350元，年均增长8%；农民人均纯收入6160元，年均增长8%；社会消费品零售总额273亿元，年均增长12%；全社会固定资产投资五年累计达到1015亿元，年均增长10%；城镇登记失业率控制在4%以内，人口自然增长率控制在6‰以内，每万元生产总值能耗控制在1吨标准煤以内。

为此，新一届政府要注重抓好以下五个方面工作：

（一）推进产业优化升级，实现经济又好又快发展

发挥临港优势，着力构建产业集约、集聚发展的园区平台，推进“四地一中心”建设，培植一批具有竞争力的支柱产业集群和特色优势产业。转变经济增长方式，增强自主创新能力，提高资源利用效率，促进经济结构优化和产业升级，使产业特色更加突出，核心竞争力明显增强。到2011年，全市工业增加值达到136.8亿元，农业增加值达到34.2亿元，旅游总收入达到82.5亿元,三次产业比重为6.1:29.2:64.7，人均GDP达到26600元，综合实力居全国大中城市中等水平；高新技术企业达到135家，高新技术产业产值达到230亿元，科技进步对经济增长的贡献率达到56%。全方位开放格局基本形成，全市外贸出口年均增长12%以上，外商直接投资年均增长10%以上。

（二）推进宜居城市建设，加快城市化进程

围绕加快城市化进程和温馨宜居城市建设，以创建全国文明城市为抓手，以“八大海口工程”为核心，高标准抓好城市规划、建设和管理，从整体上提升城市的品位，使海口成为环境特色突出、温馨宜居并充满活力的滨海城市。到2011年，“中强、西拓、东优、南控”的城市发展格局基本成型，城市建成区面积达到123.6平方公里，人口达到129万人，城市化率达到66%；城市建成区绿化覆盖率达到41%，人均公共绿地达到12平方米，全市森林覆盖率达到41%，努力建成“国家森林城市”；城市生活垃圾无害化处理率达到96%以上，城市污水集中处理率达到81%以上；城市信息化综合指数居省会城市中上水平，社会发展水平总指数位居全国前列。

（三）推进新农村建设，促进城乡协调发展

以创建文明生态村作为社会主义新农村建设的综合载体，认真落实市委、市政府《关于推进社会主义新农村建设的实施意见》，大力发展现代农业，促进农业稳定发展，农民持续增收；推动城市基础设施向农村延伸，城市公共服务向农村扩展，力争实现新农村建设阶段性目标。到2011年，全市73%以上的自然村建成文明生态村；建立起6个特色产业带，增加值达到29亿元；畜牧业增加值占农业增加值的比重达到45%，林业增加值比重达到6%，渔业产值比重达到14%,特色农产品加工转化率达到35%；中心镇建设取得阶段性成果，全市信息化服务设施加快完善,实现广播、电视、网络“村村通”。

（四）推进社会事业发展，全民共享发展成果

坚持以人为本，加快发展科技、教育、文化、体育、卫生等社会事业，满足人民群众日益增长的文化精神生活需求。始终把群众利益放在首位，重视改善人民群众基本生活，着力解决就业、住房、教育、社会保障等与人民群众生产生活密切相关的问题，努力消除社会不公平现象，切实把维护人民群众的利益落实到发展的各个方面，让人民群众共享经济社会发展和改革开放成果，加快实现和谐海口的目标。到2011年，财政对社会事业总投入达到14.6亿元，占地方财政总收入的20%，科技、教育、文化、体育、卫生等公共基础设施基本完善，全市基本普及高中阶段教育，创办“海口大学”，加快发展高等教育，争取80%以上高中毕业生能够接受高等教育；建立比较完善的社会救助体系和全民医保体系,努力扩大城乡社会保障覆盖面；城市符合条件的中低收入家庭能够买得起基本住房，困难家庭有房住。

（五）推进体制创新，增强社会发展活力和政府公共服务能力

加大改革创新力度，深化行政管理体制、文化体制、产权制度等事关我市发展全局的重点领域和关键环节的改革，打造特区省会城市体制特色，进一步增强经济社会又好又快发展的内在动力和活力。树立科学、和谐的行政理念，强化行政人员惠民利民的服务意识，不断提高政府依法行政、民主行政、科学行政的能力，着力打造公共服务型政府。到2011年，适应构建和谐社会要求的体制、机制基本建立，国有经济战略性调整基本完成，多元经济全面发展。行政流程合理科学，惠民、利民、便民的行政管理机制基本形成，实现向公共服务型政府转型。

三、2007年的重点工作

今年我市经济社会发展的主要预期目标是：全市生产总值增长11%以上；财政总收入增长13%以上；地方财政收入增长15%以上；全社会固定资产投资增长15%以上；城镇居民人均可支配收入增长8%以上，农民人均纯收入增长8%以上；城镇登记失业率控制在4%以内；人口自然增长率控制在8‰以内。

今年，我们着力做好以下七项重点工作：

（一）着力解决民生问题

今年，把解决民生问题作为政府工作的“一号工程”，要将公共资源的配置向社会事业和公共事业倾斜，保质保量地完成为民办实事项目。安排解决民生问题的专项资金6.2亿元，比去年增长59％。

完善就业服务体系，扩大社会就业。积极拓宽就业渠道，投入900万元,加强就业再就业工作,力争全年城镇新增就业人员3万人，其中下岗失业人员再就业1万人。通过大力扶持非公经济尤其是中小企业发展，努力扩大就业容量，实现经济发展和扩大就业良性互动。完善以市场为导向的就业机制，深化劳动就业制度改革，逐步形成城乡统一的人才劳务市场。建立覆盖城乡的就业服务体系，规范发展就业服务机构，强化就业指导与服务。继续落实小额担保贷款等多种优惠政策，统筹做好城乡新增劳动力就业和下岗失业人员再就业工作,积极帮助零就业家庭和就业困难人员就业。建立城乡平等的就业制度，以加快城镇化建设为契机，有针对性地组织开展职业技能培训和引导性培训，多渠道解决失地农民和农村劳动力转移就业问题，力争全年农村劳动力转移就业1.2万人，其中向省外输出5000人。

健全社会保障制度，保障群众基本生活。要依法加大社会保险费征缴力度，切实加强社会保险基金的监督管理，确保企事业离退休人员养老金按时足额发放及各项保险金依法支付。计划投入4000万元，调整提高城市低保标准和五保户供养标准。建立和完善农村养老保险制度，做好被征地农民的社会保障工作。投入资金1500万元，把符合条件的农村困难群众全部纳入低保范围。完善社会救助体系，建立健全对特困户、灾民等生活困难群众的救济机制，加大对农民工医疗救助的力度，加强城市生活无着落的流浪乞讨人员的救助管理。计划安排150万元，用于困难群众的法律援助，增强困难群众依法维权的能力。

坚持优先发展教育，促进教育公平。计划投入资金3亿元用于发展教育事业，将农村义务教育全面纳入公共财政保障范围。整合学校资源，改建和扩建19所中小学校，增加学位6000个。要将城市教育附加费、择校统筹费和农村税费改革后转移支付的资金，优先安排用于城区薄弱学校和农村学校改造，加快10个中心镇中学和中心小学的办学标准化建设。开展“教师对口支援计划”和“教师继续教育计划”，使城市公共教育资源向农村倾斜，促进城乡教育均衡发展。继续落实“两免一补”政策，对全市义务教育阶段学生免除杂费，对全市义务教育阶段公办学校在校生免除课本费，补助义务教育阶段特困家庭子女寄宿生活费，最大限度地保障城乡低收入者、残疾人和农民工子女接受义务教育。以就业为导向发展职业教育，力争全年中职教育招生规模扩大到1万人。

加强医疗卫生服务体系建设，提高群众健康水平。启动全民医保计划，争取在全国省会城市率先建立覆盖全市城乡居民的全民医保制度。加强农村三级卫生服务网络和以社区卫生服务为基础的新型城市卫生服务体系建设，为群众提供安全、有效、方便、价廉的公共卫生和基本医疗服务。安排专项资金，建设市医疗紧急救助中心，推进镇卫生院的改建、扩建工程和镇急救站建设。投入500万元,建立城乡医院对口支援、大医院和社区卫生机构双向转诊的医疗体系，优化配置城乡医疗资源。整顿药品生产和流通秩序，建立新型药品采购供应制度，解决“看病贵”和医药费用增长过快的问题。计划投入360万元，开展大病救助，确保困难群众患病时得到及时救治。继续完善农村新型合作医疗制度，使农民的参合率保持在95%以上。

（二）着力发展优势特色产业

进一步加快园区建设，推动园区经济发展。坚持把园区建设放在更加突出的位置，完善海口药谷二期、狮子岭飞地工业园的基础设施建设，力促入园项目尽快动工；做好海马汽车工业园二期配套设施建设，推动海汽15万台发动机项目建成投产和14家配套企业顺利落户，力争汽车制造业实现年产值105亿元；加快药谷二期建设，推动天煌制药等项目建成投产。积极引导和扶持园区外的工业企业向园区聚集，优化产业布局。安排工业发展基金5500万元和高新技术产业发展资金500万元，重点扶持汽车制造、生物制药、化纤纺织、食品饮料、热带农副产品深加工等一批竞争力较强的优势产业。加快推进临港工业园和物流园区建设的前期工作，大力发展临港产业，以港兴市。

积极发展现代农业，促进农村经济发展。突出“无疫区”、“无公害”特色，继续调整优化农业结构和布局，重点发展以生猪和文昌鸡饲养为主的畜牧业，以热带水果、瓜菜、花卉苗木为主的种植业，以及以养殖罗非鱼为主的渔业等农业特色产业。安排资金500万元，扶持出口型农业发展。启动优势产业出口示范区（基地）建设，建立现代生态畜牧出口示范区，建成一批文昌鸡、水产品、热带水果出口基地、种苗基地、冷藏加工基地和农村新型标准化养殖小区；安排1200万元，推进大中型沼气池建设，积极发展生态循环农业和设施农业。完善农业科技110服务体系，大力发展电子农务，提高农业信息化水平。加快制定和实施优势农产品质量安全标准和生产技术规程，扩大无公害农产品、绿色食品生产，健全农产品检测网络，确保农产品质量安全。

大力发展消费型服务业。高度重视服务业在促进生产、拉动消费、扩大就业、增加地方税收等方面的作用，加大政策扶持和推动力度，重点支持消费型服务业发展。完成《海口市旅游业发展总体规划》，推进海南游客到访中心、自驾车营地等11项旅游重点工程建设；加强旅游宣传促销，不断提升海口知名度；精心组织开展节庆会展活动，举办第五届中国（海口）国际旅游商品交易会、《城市之间》国际版活动和第八届中国海南岛欢乐节海口系列活动。努力提高城乡商业发展水平，以商业网点规划为指导，发展优化海秀东路、国贸等商业圈，改造提升府城中心区、解放西路、新华南路老商业区，结合城区的拓展，开发新的特色商业街；大力发展现代物流业，引导连锁经营从超市、百货店向便利店、专卖店等业态渗透；抓住农村商品流通改革试点的机遇，扎实推进“万村千乡”市场工程和“农村电子商务信福工程”。培育和扩大住房新的消费热点，合理确定供地规模，完善住房供应体系，优化商品房结构，满足不同层次的住房需求；加快廉租房和经济适用房制度建设，采取“政府主导、社会投资、市场化运作”的模式，建设1000套安置房，解决旧城区拆迁户和城市特困居民的住房问题，促进房地产业健康有序发展。积极发展信息咨询等服务业，规范物业管理、康体保健和家政等便民利民服务，支持金融保险、邮电通讯等产业发展，努力满足人民群众生产生活需要。

（三）着力推进温馨宜居城市建设

加强城市规划建设，提高城市品质。认真贯彻落实我市《关于加快城市化进程的若干意见》，继续做好规划编制工作，重点抓好府城、长流等6个镇并入主城区的规划和城郊结合部的20个示范村庄的建设规划。制定和完善《海口市控制性详细规划编制与管理办法》、《海口市村镇规划管理办法》等。启动美丽沙、新埠岛、西海岸、大英山老机场和桂林洋高校区等片区建设，推动长流片区路网、市行政中心等项目建设，优化城市西部资源，拓展城市发展空间。实行旧城改造政府“零收益”政策，积极引进社会投资，推动海甸溪北岸、“一园两湖”、玉沙村等旧城改造项目。推进秀英港二期等续建工程，促进东环铁路、新海滚装客运港等项目开工建设。实施绕城公路、滨江西路、海甸岛环岛路、货运大道二期等城市道路建设工程和人民桥、新埠桥改造工程。安排专项资金，改造和平大道、白龙路等5条主次干道的街景立面,继续实施小街小巷路面硬化建设和道路的路灯安装工程。

创新城市管理机制，提升城市管理水平。建立健全市、区、街道（镇）分级管理体制，实行“数字化、网络化、下沉式”管理，充分发挥社区和居委会在城市管理中的积极作用，努力构建社会协同、公众参与的城市管理新格局。启动数字化“万米网络”管理和“12319”热线，抓好执法队伍建设，塑造文明执法新形象。继续推进违法建筑、交通秩序、市场秩序等专项整治，力争拆除违法建筑30万平方米。制定《海口市优先发展公交实施意见》，大力发展城市公交，改善市民出行条件。安排860万元，实施候车亭改造。

加强环境建设保护，建设“生态”海口。动员全市力量，抓紧解决影响我市市容市貌等城市环境的重点问题，进一步提高城市环境质量，以优异成绩迎接国家环保模范城市复查。加大环境保护力度,严厉打击乱采、乱挖、乱伐、乱排放行为。整治城区水系，改善地表水体的水质。强化环境卫生综合治理，加强建筑垃圾监管，做好生活垃圾收集及转运。安排资金1000万元，完成颜春岭垃圾填埋场垃圾渗透液处理工艺改造，新建一批垃圾转运站。积极做好《海口市森林生态体系总体规划》、《海口市城区绿线控制性规划》等城乡森林、绿地规划。实施“百里绿色长廊”乡村公路林、海防林建设和南海大道、琼州大道等城市道路绿化改造工程，提高城乡道路绿化水平。加快推进双拥公园、凤翔公园、白沙门公园、秀英公园和城市街心街边公园绿地以及城郊“森林化”、乡村“生态化”建设，力争全年造林2667公顷，努力把海口建设成为环境优良的生态城市。

（四）着力推进社会主义新农村建设

认真贯彻落实中共中央、国务院《关于积极发展现代农业，扎实推进社会主义新农村建设的若干意见》和我市《关于推进社会主义新农村建设的实施意见》，坚持工业反哺农业、城市支持农村和多予少取放活的方针，大幅度增加对“三农”的投入，全年安排“三农”资金2.67亿元，着力促进农业增效、农村发展、农民增收。

大力发展镇域经济。继续巩固、完善、加强支农惠农政策，健全农业支持补贴制度，建立农业风险防范机制。鼓励农民投资现代农业，大力发展特色农业，扶持农业产业化龙头企业发展。抓好村镇规划，通过规划引导、政策支持、示范带动等办法，支持“一村一品”经济发展。引导和扶持有条件的村镇发展“农家乐”和旅游产品加工、服装加工等非农产业。加大中心镇的建设投入，支持每个中心镇1000万元启动资金，逐步完善中心镇的公共服务设施，为镇域经济发展创造基础条件。

加大对农业农村基础设施建设的投入。搞好水库大坝除险加固，继续实施中型水库干渠道和小型水库干渠的防渗配套工程。实施千亩以上田洋整治项目,完成田洋整治10个，面积达5万亩。投资2100万元，完成二期15栋防洪楼建设。大力实施农村公路通畅工程，投资6000多万元,完成总长228公里的农村公路建设，改造危桥9座，解决农民行路难问题。继续加强农村饮水、用电、通信等基础设施建设，努力改善农村生产生活条件。

深入开展文明生态村创建活动。按照优化生态环境的要求，围绕打造田园海口的目标，突出热带海岛都市村镇特色，加大环境综合整治力度，搞好庭院绿化美化，改善村容村貌。积极推广“区片联创”经验，建立以城带乡、城乡共建的创建机制，不断扩大文明生态村覆盖面，力争今年全市文明生态村总数达到980个，占自然村总数的44%。

（五）着力推进改革开放和科技创新

创新管理体制，优化发展环境。继续深化行政管理体制改革，按照财权和事权相一致的原则，根据省、市确定的职责，把能下放的管理权限尽量下放到区，实行管理重心下移；实施机关事业单位工资收入分配制度改革，规范国家公务员津贴补贴。加快财政体制改革，按照构建公共财政体系和实现公共服务均等化的要求，调整市、区财政管理体制，做到财权与事权相匹配；优化财政支出结构，保证政府资金更多地投向公共基础设施、生态环境、社会事业等领域；建立财政资金绩效评价机制，提高财政资金的使用效率。加快政府投融资体制改革，拓宽市政公用事业和城市基础设施的融资渠道，推进市场化进程。抓好文化体制改革试点单位的改制工作，推进文化事业和文化产业发展。进一步完善国有资产管理体制改革，强化国有资产的监督管理；完成水务集团的招商合作，积极扶持港航控股公司等国有企业做大做强；运用拍卖、股份合作等多种方式，进一步加快劣势国有企业退出步伐。建立担保平台，进一步解决非公经济融资难问题，加快非公经济规模的形成和核心竞争力的提高。

深化科技体制改革，提高科技创新能力。继续加大科技创新力度，广泛开展与国内外高校和科研院所的科技交流与合作，大力引进高新技术项目。完善科技创新体系，加快利用高新技术提升传统产业，以及培育5家市级技术创新示范企业和5家省级企业技术研发中心，提高企业自主创新能力。调整科技经费投向，重点向生物医药、生态环保、电子信息、海洋开发和农业技术推广等领域倾斜。加强知识产权保护和培育，鼓励企业运用知识产权，增强市场竞争能力。继续实施海口“十一五”人才发展规划，做好优秀人才的培养、引进和使用工作。

进一步加大对外开放力度。通过组建海口市工业和招商局，明确招商主体，完善招商政策，加强招商引资工作。重点推进产业开放和抓好招商引资，突出“招大引强选优”原则，积极引企业、上项目。充分利用会展、互联网等各种平台，加大园区推介力度，引进工业项目向狮子岭“飞地工业”园和药谷二期园区集聚。加强招商基础性工作，及时收集、筛选、储备一批项目，完善招商库功能，为各类招商提供服务。继续做好签约项目的跟踪协调服务，帮助企业解决存在的问题，积极推进项目加快落实。进一步做好外事侨务工作，加强与国内外经济、文化、科技等方面的交流。

（六）着力推进文化建设和政治建设

积极发展和谐文化，丰富群众精神生活。继承和发展优秀传统文化和民族文化，推进中国历史文化名城建设。制订文物保护规划，加强文物保护，做好中山路等五条具有南洋建筑风格商业街的保护和利用。计划投入4772万元加快文体事业的发展，加强社区文体场所和设施建设，建设区级图书馆，为社区和村镇配备一批健身器材。搞好群众性文体活动，办好冼夫人文化节、琼剧文化节等品牌文化活动，引进国内外专业院团来海口举办精品文艺演出，深入开展广场文艺活动和全民健身运动，力争办好我市首届农民运动会。继续实施文化精品工程，创作一批具有海口特色的文化精品，努力培育书报刊、文艺演出等文化产品市场。

推进民主法制建设，完善民主政治。严格执行人大及其常委会的决议决定，自觉接受人大及其常委会的法律监督和人民政协的民主监督，广泛听取民主党派、工商联、人民团体、无党派人士和社会各界的意见，主动邀请人大代表评议政府工作。积极贯彻国务院《全面推进依法行政实施纲要》，加强和改进立法工作，针对我市经济社会发展的重点难点问题，积极向人大提出立法建议，坚持依法行政，推进依法治市进程。贯彻落实《海南省深化乡镇政务公开实施意见》，继续加大政务公开、厂务公开、村务公开的力度，充分发扬基层民主，拓宽社情民意的表达渠道，搭建多种沟通平台，切实保障人民群众的知情权、参与权、监督权。

强化社会治安，维护公共安全。加大对公共安全的投入，大力开展平安创建活动，以开展“两抢一盗”专项整治为重点，严厉打击各种刑事犯罪，力争刑事发案率同比下降3%，其中“两抢”案件发案率同比下降10%。加大禁毒工作力度，研究戒毒工作新模式，全力抓好禁毒工作。推行食品安全准入制度和强制认证制度，加强食品、药品、餐饮卫生监管，杜绝不合格的产品进入市场。抓好安全生产，严格安全生产问责制，重点开展道路交通、消防安全、建筑市场等领域的安全生产整治，遏制重特大事故发生。开展危房普查，控制危房使用，做好有序改造。完善应急管理体制，建立公共危机防控体系，加快应急信息平台建设，提高危机处理和抗风险能力。认真落实各项优抚政策，创新军民共建模式，构建和完善国防动员体系。

（七）着力提高政府构建和谐社会的能力

改进公共服务方式，打造服务型政府。创新公共服务体制，把政府的主要职能、主要财力集中到为老百姓提供更多、更好的公共产品上，提高政府自身提供公共服务的能力。以发展社会事业和解决民生问题为重点，调整和优化政府公共服务结构，合理配置公共资源，增加社会性公共服务支出，将财政支出的重点转向公共教育、社会保障、公共卫生、公共安全和公共基础设施等方面。建立长效的行政管理机制，充分调动机关工作人员的积极性和创造性，营造实干、创新的工作氛围。强化全局观念和责任意识，加强各部门的协调、沟通。建立健全科学有效的利益协调机制、社会纠纷调处机制和权益保障机制，增强预防和化解矛盾的能力。加强领导干部作风建设，大力发扬求真务实、奋发进取的精神，把更多的时间和精力用于深入基层、调查研究、解决问题上。

加强政府廉政建设，严厉惩处腐败行为。认真贯彻落实中纪委七次全会精神和胡锦涛同志在七次全会上的重要讲话精神，抓好《中共海口市委关于贯彻落实〈建立健全教育、制度、监督并重的惩治和预防腐败体系实施纲要〉的具体意见》的落实，坚持标本兼治、综合治理、惩防并举的反腐倡廉方针，按照市委的统一部署，加强廉政文化建设，抓好思想道德教育和党纪国法教育。进一步完善惩治和预防腐败体系，严格执行领导干部经济责任审计、重大事项报告等制度，加强对制度执行情况的督促检查。严肃查处侵害群众利益的各类违纪违法案件，对损害我市投资环境和利用工作之便捞取好处的行为，必须进行问责,严重的要依法惩处，以廉洁、勤政、高效的作风，为海口人民行好政、服好务。

各位代表！海口未来五年的发展方向已经明确，新一届政府将认真贯彻落实市第十一次党代会和本次人代会精神，团结和依靠全市人民，同心同德，求真务实，艰苦创业，锐意进取。我们有理由相信，经过五年的奋斗，一个繁荣富裕、环境优良、社会和谐的海口将呈现在我们面前！
